# *KvLEA*, a New Isolated Late Embryogenesis Abundant Protein Gene from* Kosteletzkya virginica* Responding to Multiabiotic Stresses

**DOI:** 10.1155/2016/9823697

**Published:** 2016-03-30

**Authors:** Xiaoli Tang, Hongyan Wang, Liye Chu, Hongbo Shao

**Affiliations:** ^1^Key Laboratory of Coastal Biology & Bioresources Utilization, Yantai Institute of Coastal Zone Research (YIC), Chinese Academy of Sciences (CAS), Yantai 264003, China; ^2^University of Chinese Academy of Sciences, Beijing 100049, China; ^3^Yantai Academy of China Agricultural University, Yantai 264670, China; ^4^The Central Laboratory, Jiangsu Academy of Agricultural Sciences, Nanjing 210014, China; ^5^Jiangsu Key Laboratory for Bioresources of Saline Soils, Provincial Key Laboratory of Agrobiology, Institute of Agro-Biotechnology, Jiangsu Academy of Agricultural Sciences, Nanjing 210014, China

## Abstract

The LEA proteins are a kind of hydrophilic proteins, playing main functions in desiccation tolerance. However, their importance as a kind of stress proteins in abiotic stress is being clarified little by little. In this study we isolated, cloned, and identified the first* KvLEA* gene in* Kosteletzkya virginica*. Bioinformatic analysis showed that the protein encoded by this gene had common properties of LEA proteins and the multiple sequences alignment and phylogenetic analysis further showed that this protein had high homology with two* Arabidopsis* LEA proteins. Gene expression analysis revealed that this gene had a higher expression in root and it was induced obviously by salt stress. Moreover, the transcripts of* KvLEA* were also induced by other abiotic stresses including drought, high temperature, chilling, and ABA treatment. Among these abiotic stresses, ABA treatment brought about the biggest changes to this gene. Collectively, our research discovered a novel LEA gene and uncovered its involvement in multiabiotic stresses in* K. virginica*. This research not only enriched studies on LEA gene in plant but also would accelerate more studies on* K. virginica* in the future.

## 1. Introduction

Plants are continuously subjected to environmental stresses such as drought, high salinity, cold, chilling, and freezing along their life cycles. These abiotic stresses can negatively affect plants in growth and development, leading to enormous losses in agricultural production throughout the world [1, 2]. For survival, plants have developed a variety of anatomical and morphological features which may alleviate the damage of these adverse conditions. Both the activation of stress responsive genes and the synthesis of resistant proteins trigger a series of physiological and metabolism responses to environmental stresses [3–5]. The late embryogenesis abundant (LEA) protein is one of the many resistant proteins which were synthesized under adverse conditions [6, 7].

The researches on LEA proteins began with the discovery of the first LEA protein, which was discovered in cotton seeds more than three decades ago [8]. From then on, the studies on LEA proteins have never stopped. So far, we have known that these proteins are highly hydrophilic and intrinsically disordered, with low proportions of cysteine and tryptophan and high proportions of glycine, glutamate, lysine, and threonine [9, 10]. According to the amino acid sequences and conserved motifs, the LEA proteins are generally categorized into different groups in different plants [11]. For instance, there are as many as 51 LEA proteins in* Arabidopsis* and they are divided into nine groups, including LEA-1, LEA-2, LEA-3, LEA-4, LEA-5, SMP, PvLEA18, dehydrin, and AtM [7]. However, according to the PFAM database, LEA proteins were grouped into eight families: dehydrin, LEA_1, LEA_2, LEA_3, LEA_4, LEA_5, LEA_6, and SMP, without the AtM group [12]. The LEAPdb which was a public database of LEA gene sequences gave a more detailed classification, with 12 nonredundant groups [13].

As the researches on LEA move along, the importance of LEA proteins is reported by more and more studies. Their synthesis in large amount during the late stages of seed development enables the maturing seeds to acquire the desiccation tolerance. Importantly, by means of stabilizing proteins, nucleic acids, and cell membranes, LEA proteins are proved to be the common components associated with tolerance to various stresses [14, 15]. For example, the maize LEA3 gene was discovered to be induced by ABA and hyperosmolarity [16], and afterwards it was proved to be able to reduce cell shrinkage effects under dehydration [17]. Similarly, a novel LEA gene was identified in wheat which was induced by freezing tolerance [18]. The reduced expression of CeLEA1, a LEA-like protein from* Caenorhabditis elegans,* led to reduced survival under desiccation and osmotic and heat stresses [19]. On the other hand, ectopic expressions of LEA in various organisms also gave the evidence for their functions in stress protection [9, 20, 21]. The transgenic wheat and rice with heterogeneous expression of LEA gene from barley acquired enhanced drought and salt tolerance [22, 23]. Overexpressing NtLEA7-3 in* Arabidopsis* endowed the transgenic plants with enhanced resistance to cold, drought, and salt stresses [24]. In* Arabidopsis*, the SAG21 was discovered to enhance the tolerance against bacterial pathogen (Pst DC3000) and oxidative stress [25]. The LEA protein Rab28 which is localized in the nucleus in maize embryo can enhance transgenic maize tolerance to drought stress [26]. Therefore, the LEA proteins are involved not only in water deficit but also in abiotic stresses. It is one of the key factors for plants to respond to adverse environment.

*K. virginica* is a newly introduced species due to its amazing salt stress resistance and potential economic value [27]. It was reported that this plant was able to maintain normal growth and development under adverse conditions with 0.3% to 2.5% sodium salt [28]. Thus, it must possess highly efficient physiological, biochemical, and molecular mechanisms to do this. Therefore, we are interested in its special mechanisms under salt stress, in the hope of uncovering novel resistance mechanisms in it. However, the genetic information on it is very scarce. As far as we know, there are almost no reports about the molecular mechanism of salt resistance on* K. virginica*, except for one report covering the gene expression analysis on* K. virginica* seedlings by cDNA-amplified fragment length polymorphism (cDNA-AFLP) technology [29].

Based on the diverse biological functions of LEA proteins as stated before, we wondered whether LEA also had similar roles in* K. virginica*. Therefore, this study aimed to clone, isolate, identify, and explore the possible functions of a LEA gene in* K. virginica*. With the help of transcriptome analysis of* K. virginica* under salt stress in our recent research, we got the core sequence of the gene [30]. RACE technology was adopted to clone the whole sequence of this gene. The biological software such as ProParam, InterProScan 5, BLASTp, and CLUSTALWX was used to do the sequence prediction and analysis. Phylogenetic tree was constructed to analyze which subgroup it may belong to. Furthermore, qPCR assays were carried out to detect the gene expression patterns under abiotic stresses. This study about the first LEA gene in* K. virginica* further enriched the research information on LEA gene. Meanwhile, it also laid a good foundation for molecular studies in* K. virginica*.

## 2. Material and Methods

### 2.1. Plant Materials and Growth Conditions

The seeds of* K. virginica* used in this study were coming from the* K. virginica* growing in Yellow River Delta, Shandong Province, China. The collected seeds were disinfected with 70% alcohol solution for 30 s and 0.1% mercuric chloride solution for 10 min and then washed thoroughly with sterile water six times. The surface-sterilized seeds were laid on wet filter paper for germination and then the germinating seeds were planted in vermiculite in plastic pots watered with Hoagland's solution [31]. The seedlings were cultivated under conditions of 16 h light/8 h dark and the temperature was kept at 25°C/20°C, respectively, in day and night in artificial climatic chambers (Huier, China). The humidity was kept at 60% and the seedlings were watered every 3 days with nutrition solution. Two-week-old and homogeneous seedlings were chosen for different samplings and six homogeneous seedlings at least were contained in each sample. However, the samples for tissue expression pattern detection came from the different corresponding tissues, respectively. All the samples were frozen with liquid nitrogen and stored at −80°C.

### 2.2. Cloning and Sequence Analysis of* KvLEA*

For gene cloning total RNA was extracted with RNAiso Plus (TaKaRa, total RNA extraction reagent) according to the operation steps. Agilent 2100 BioAnalyzer (Agilent Technologies, CA, USA) was used for the detection of the RNA quality and quantity. The full length of* KvLEA* was amplified by RACE according to the user manual (SMART*™* RACE cDNA Amplification Kit). The gene-specific primers (GSPs) (forward: CCTCCTGCTCCTGGCTCTTA; reverse: GGTGGCTGAAGGGACGGAGTA) and the nested primers (NGSPs) (forward: CCTGGCTCTTACTACCAACGAC; reverse: AAGCAGACGGCATCCGAAAC) were designed based on the cDNA core sequence coming from our previous transcriptome sequencing data, which had been deposited in Transcriptome Shotgun Assembly (TSA) Sequence Database with the accession number GCJL00000000 [30]. The PCR products were cloned into pEASY®-T5 Zero Cloning Vector (Transgene, China) after purification from gels for sequencing. The physical and chemical parameters of KvLEA protein were analyzed with ProParam, InterProScan 5 (http://www.ebi.ac.uk/Tools/pfa/iprscan5/), and the Sequence Manipulation Suite (http://www.bio-soft.net/sms/). The BLASTp was performed at NCBI (National Center for Biotechnology for Information, http://blast.ncbi.nlm.nih.gov/Blast.cgi) [32]. Multiple sequence alignment was carried out among the deduced KvLEA protein and all the eighteen LEA_4 proteins in* Arabidopsis* with CLUSTALWX program. The phylogenetic tree was produced based on the multiple sequence alignment using MEGA 6.0 software by Neighbor-Joining method [33].

### 2.3. Multiabiotic Stress Treatments

The two-week-old* K. virginica* seedlings grown in plastic pots (six seedlings at least in each pot) were selected for our research. For heat and chilling stress treatments, the homogeneous seedlings were transferred to different artificial climatic chambers (Huier, China) at 42°C and 4°C, respectively. For high salinity, drought, and ABA stress treatments, the plants were irrigated with nutrient solution with 300 mM NaCl, 15% PEG 6000, and 100 *μ*M ABA, respectively. The handling times for all the treatments were 0, 2, 6, 12, and 24 hours. The control sample was kept at normal growth conditions and irrigated with nutrition solution. The control and treated samples were all removed from the matrix and washed carefully after treatments. Then, the seedlings were soaked up cautiously and quick-frozen in liquid nitrogen and stored at −80°C for use.

### 2.4. RNA Isolation and Gene Expression Analysis

The total RNA was extracted from samples with RNAiso Plus (TaKaRa, total RNA extraction reagent). The first-strand DNA was synthesized with TransScript® One-Step gDNA Removal and cDNA Synthesis SuperMix (TransGen, China). The qPCR was performed with the cDNA diluted at a certain ratio as template and the gene-specific qPCR primers were designed with the primer-design software Beacon Designer 7.0. The primers are 5′-TAGTAAGAGCCAGGAGCAGGAG and 3′-TTGAACTCGCCAGCCTTATCTC. The primers of the internal control gene* Kv*EF1-*α* are 5′-TCAATGAGCCAAAGAGG and 3′-CAACACGACCAACAGGA.

The qPCR was conducted on the instrument ABI Prism® 7500 FAST (Applied Biosystems, Foster City, CA) and the reaction was performed using SYBR® Green Real-Time Selected Master Mix (Applied Biosystems by Life Technologies®) according to the user manual. The reaction volume was 20 *μ*L, including 2 *μ*L diluted cDNA template, 10 *μ*L 2x SYBR Master Mix, and 200 nM of each primer. The reaction processes were as follows: (1) initial denaturation at 95°C for 2 min, 40 cycles of 15 s at 95°C for denaturation of template, and 1 min at 60°C for annealing and extension. The detection of the fluorescence signal was conducted at the temperature between 60°C and 90°C. The primer specificity was ensured beforehand by the typical melting curve and amplification plot as well as the sole product in RT-PCR. The cDNA template was diluted to guarantee that the Ct values of all the samples were located in the appropriate scope. Each sample was performed in triplicate and three biological replicates were performed to decrease the error.

### 2.5. Statistical Analysis

The relative expression level was calculated with 2^−ΔΔCt^ method. Namely, the ΔCt values were the difference of the Ct values between target gene and the internal control gene (ΔCt = targetCt − EFCt). ΔΔCt values were the difference of the ΔCt values between sample ΔCt and control ΔCt (ΔΔCt = sampleΔCt − controlΔCt). The mean value and standard error (SD) of the three biological replicates were calculated through Microsoft Excel 2007. Plotting was performed with SigmaPlot 12.0.

## 3. Results

### 3.1. Isolation and Sequence Analysis of* KvLEA* Gene

**Figure 1:**
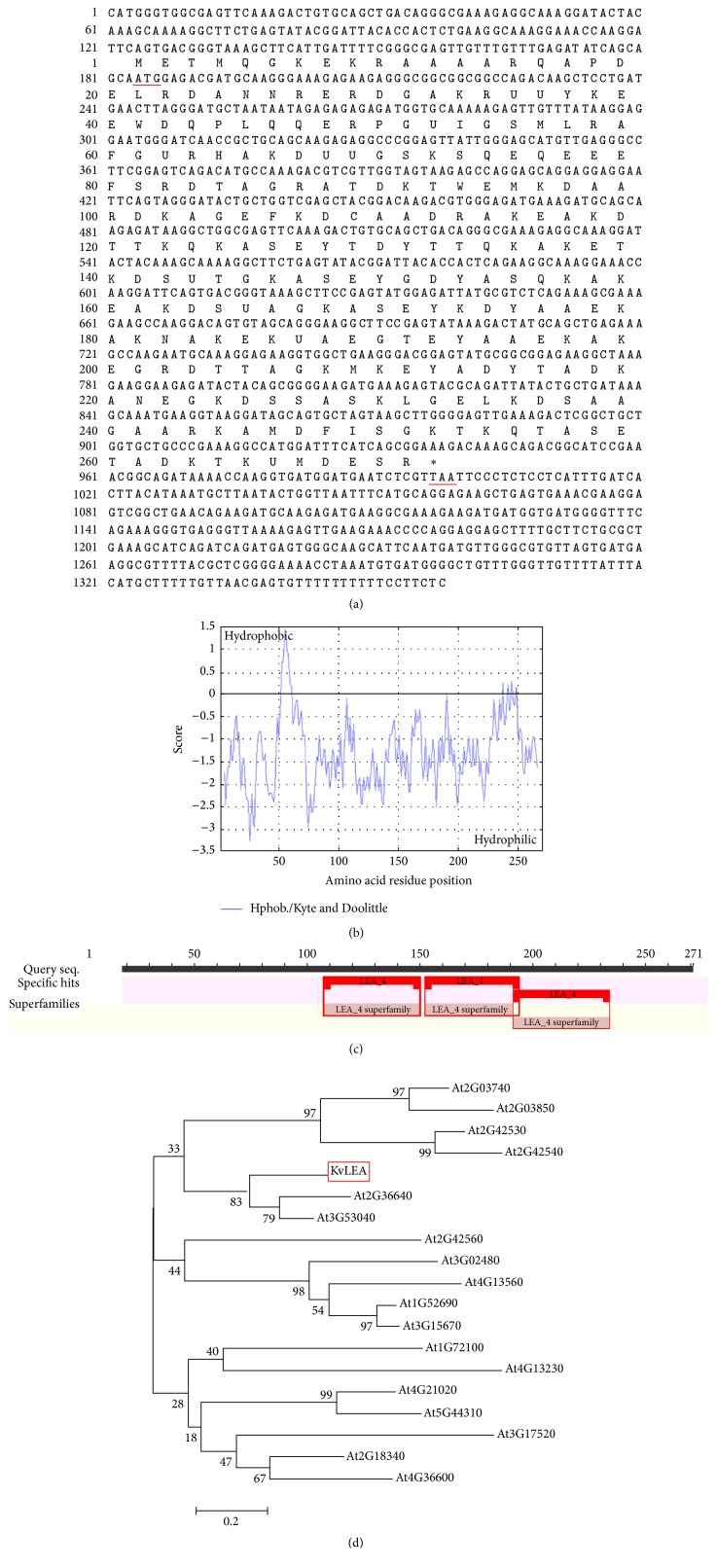
Sequence, hydrophilic, repeat motifs and phylogenetic analysis of* KvLEA*. (a) The nucleotide and predicted amino acid sequences of* KvLEA* gene. The initiation codon and termination codon were highlighted with red underline. The encoded amino acids were listed correspondingly to the ORF. (b) Hydrophilic analysis of* KvLEA*. The analysis was conducted according to Kyte-Doolittle hydropathy plot. The section above zero stands for the hydrophobic part of the sequence and the section below zero represents the hydrophilic part. (c) The BLASTp search of the predicted amino acid sequence. The red boxes show the category and position of the conserved motifs (LEA_4 accession: pfam02987). (d) Phylogenetic tree of* KvLEA* and eighteen LEA_4 proteins in* Arabidopsis*. The phylogenetic tree was constructed using MEGA 6.0 by Neighbor-Joining method based on the multiple sequence alignment of the nineteen LEA proteins. The protein sequences of the* Arabidopsis* LEA genes were acquired from the Arabidopsis Information Resource (TAIR) database (http://www.arabidopsis.org/). The gene IDs and names of the proteins were listed in Supplemental S3.

The full length of* KvLEA* gene was determined by 5′ and 3′ rapid amplification of cDNA ends (RACE) technology. The total sequence of the gene is 1359 bp, along with a 184 bp 5′ untranslated region (UTR) and a 359 bp 3′ UTR. Hence, the open reading frame (ORF) of* KvLEA* is 816 bp and encodes a protein with 271 amino acids (Figure 1(a)). The sequence information of this gene had been presented to NCBI database and the GenBank accession number was KP688391. The KvLEA protein was predicted to be 29.79 kD molecular mass and the isoelectric point (pI) was 7.61. The grand average of hydropathicity (GRAVY) and the instability index of it were −1.292 and 28.49, respectively (http://web.expasy.org/protparam/). Meanwhile, the amino acid sequence of* KvLEA* protein is rich in alanine (17%), lysine (15%), and glutamic acid (11%), while lacking cysteine, histidine, tryptophan, and arginine. Thus, the percentage of the hydrophilic amino acid in it is reaching up to 70.85%, a hydrophilic protein. According to Kyte-Doolittle hydropathy plot [34],* KvLEA* protein also should be hydrophilic, except for a few hydrophobic residues at its N-terminus (Figure 1(b)).

With the deduced amino acid sequence, a BLASTp search was conducted at NCBI database (http://blast.ncbi.nlm.nih.gov/Blast.cgi). The results indicated that* KvLEA* protein contained three LEA_4 (accession: pfam02987) superfamily conserved motif repeats and the locations of the three repeat motifs were 107–150 bp (*E*-value: 4.64*e* − 05), 152–194 bp (*E*-value: 3.37*e* − 04), and 191–234 bp (*E*-value: 4.55*e* − 04), respectively (Figure 1(c)), implying that this LEA protein might belong to LEA_4 group, which was the largest group among the nine groups in* Arabidopsis*. The motif analysis by InterProScan 5 (http://www.ebi.ac.uk/Tools/pfa/iprscan5/) also showed a similar result, with four repeats of LEA_4 (accession: pfam02987) superfamily conserved motifs (Supplemental S1 in Supplementary Material available online at http://dx.doi.org/10.1155/2016/9823697). We also analyzed the transmembrane domain of the protein at the same time. The prediction indicated that it had no transmembrane domain, staying with the same character of the other LEA_4 proteins (Supplemental S2). To further verify whether* Kv*IEA belonged to the LEA_4 type LEA gene, a phylogenetic tree was constructed with the predicted sequence of* KvLEA* protein and all the eighteen LEA proteins belonging to LEA_4 group in* Arabidopsis* (Figure 1(d)). The result revealed that* KvLEA* should be a LEA_4 group member, sharing high genetic homology with two* Arabidopsis* genes At2g36640 and At3g53040 in the LEA_4 group.

### 3.2. *KvLEA* Expression Profiles under Normal and Salt Stress Treatment

**Figure 2:**
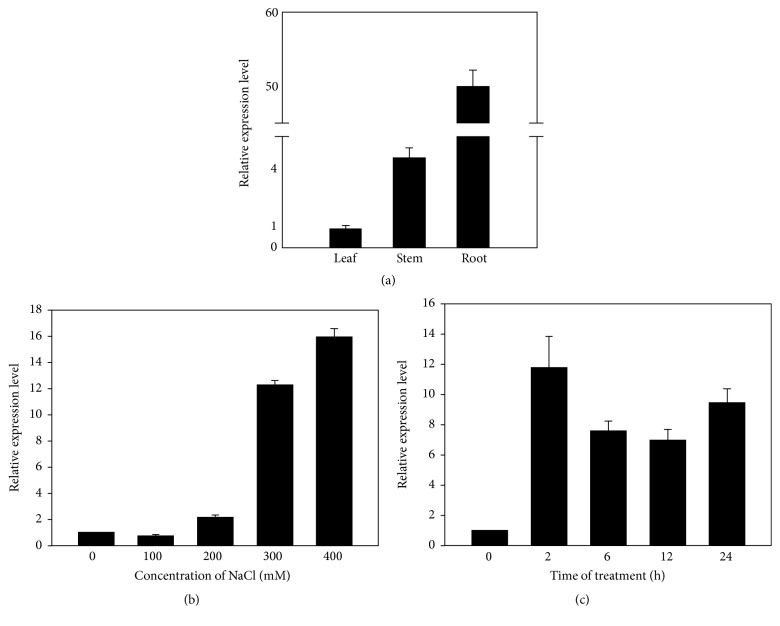
The expression patterns of* KvLEA* gene in different tissues and under salt stress. (a) The expression levels of* KvLEA* in different tissues. Root, stem, and leaf were collected from seedlings growing under normal condition and total RNA was extracted from them, respectively. The expression quantity in leaf which was the smallest among three samples was set as the reference. The expression levels of the other two were the relative expression levels compared to leaf. (b) The transcripts accumulation of* KvLEA* under different NaCl concentrations (0, 100, 200, 300, and 400 mM). The 0 mM NaCl treatment was the control sample treated with the same nutrition solution without any NaCl. (c) The transcripts accumulation of* KvLEA* at different time of salt stresses (0, 2, 6, 12, and 24 h after salt treatment). The 0 h treatment was the corresponding control sample. The samples of NaCl treatment were the whole seedlings. Three biological replicates were performed and the mean values of the three biological replicates were used for plotting. Error bar indicates standard error (SD, *n* = 3).

As soon as we acquired the full gene sequence, we determined the tissue expression profiles of this gene at first. RNA was extracted from root, stem, and leaf, respectively, and the determination of the expressions was carried out by qPCR. The expression pattern (Figure 2(a)) showed that* KvLEA* tended to be expressed in root with priority. The expression level in root is more than 50 times that in leaf, implying that it might play an important role in root.

On account that* KvLEA* gene was obtained by screening our transcriptome sequencing data (accession number GCJL00000000) [30] of* K. virginica*, which displayed apparent transcripts accumulation under salt treatment, we studied this gene under salt stress in detail. We detected the conditions of the expression under different salt stress duration and different salt concentrations. In keeping with the transcriptome results, the gene indeed was induced by salt stress in different degrees under different salt conditions. With the increase of the salt stress intensities, the responses strengthened gradually (Figure 2(b)). 300 and 400 mM NaCl led to pronounced elevations in gene expression compared with 100 and 200 mM treatments. As shown in Figure 2(b), 400 mM NaCl triggered the most pronounced surge in expression at about 16-fold increase. With regard to the different duration of salt stresses, the accumulations of* KvLEA* gene kept at a high level all the time from 2 h to 24 h (Figure 2(c)). The transcripts accumulated a lot immediately after 2 h treatment and kept steadily high in the following time. Therefore,* KvLEA* should play an important role in salt tolerance and contribute to the high salt resistance of* K. virginica*.

### 3.3. Expression Patterns of* KvLEA* under Various Abiotic Stress Treatments

**Figure 3:**
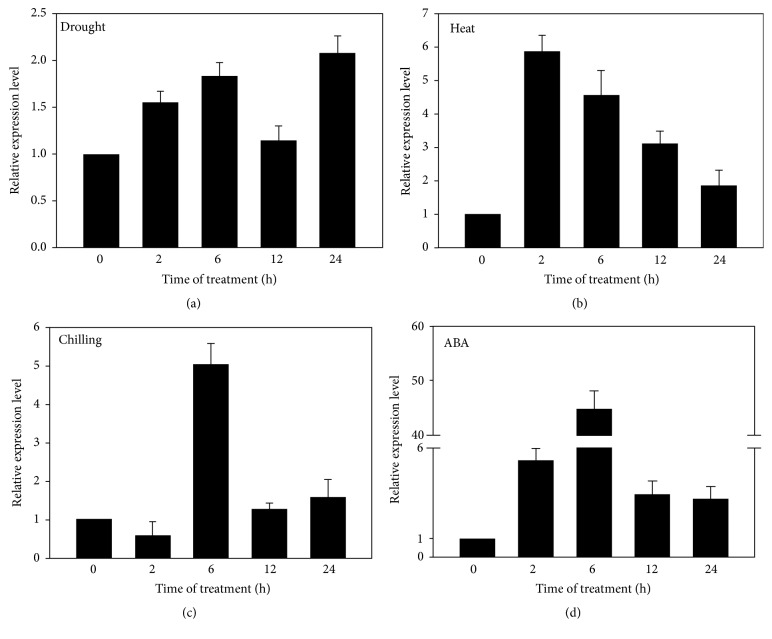
Transcript accumulation of* KvLEA* in response to different abiotic stresses. (a) 15% (w/v) PEG 6000 treatment, (b) high temperature 42°C treatment, (c) 4°C chilling treatment, and (d) 100 *μ*M ABA treatment. The heat and the chilling stresses were achieved in artificial climatic chambers. Drought and ABA treatments were performed through irrigating sufficient nutrition solutions with corresponding concentrations of PEG and ABA; meanwhile, the control sample was watered only with nutrition solution. Three biological replicates were carried out for every stress treatment and the mean values were used for the expression analysis. Expression analysis was carried out with ΔΔCt method. Error bar indicates SD (*n* = 3).

Previous researches have reported that some LEA proteins participated in various abiotic stresses such as high temperature [21], high salinity [35, 36], osmotic stress [36], and chilling stress [24]. Then, we wondered whether our* KvLEA* protein was also involved in these abiotic stresses, so we also examined the performances of* KvLEA* gene under these conditions. The results showed that* KvLEA* gene responded to drought, heat, chilling, and ABA treatments too. Under drought stress treatment, the* KvLEA* transcripts were induced and the accumulation increased progressively with time (Figure 3(a)), while the responses of* KvLEA* to drought stress were weak. The difference could be regarded as not significant or weak (generally, expression ratio ≥ 2 or ≤0.5 was deemed as the boundary of the significance). Compared to drought treatment, heat stress triggered more obvious accumulation to* KvLEA* transcripts and the accumulation appeared to be the highest at 2 h after treatment. However, contrary to drought stress, the transcripts under heat stress decreased with time (Figure 3(b)). The responses of* KvLEA* to chilling stress were interesting. It only displayed distinct response at 6 h after treatment, yet there were no differences at other time points (Figure 3(c)). In addition, 100 *μ*M ABA caused the most obvious accumulations to this gene among all the abiotic stresses, especially at 6 h with 44-fold increase (Figure 3(d)).

## 4. Discussion

To adapt to the ever-changing ambient conditions, higher plants have developed complicated resistance mechanisms along with their evolutionary history. The existence of LEA proteins is a good example. Generally, these proteins share some common properties, including high hydrophilicity, repeat motifs, and low sequence complexity [37, 38]. In the present study, we isolated and cloned the first LEA gene in* K. virginica*. In the context of previous studies, this research also analyzed the sequence information of* KvLEA* gene at first. Similar to the common properties of most LEA proteins, KvLEA protein also has high hydrophilicity and contains repeat motifs. It is composed of high percentage of hydrophilic amino acids and the percentage of the hydrophilic amino acids is as much as 70.85%. Both the value of the grand average of hydropathicity (GRAVY) and the analysis of Kyte-Doolittle hydropathy plot verify its hydrophily at the same time (Figure 1(b)). On the other hand, the BLASTp search (Figure 1(c)) and the InterProScan 5 prediction (Supplemental S1) proved the existence of the repeat motifs in* KvLEA* protein.

On account of the function researches, LEA proteins are proposed to have a broad influence on abiotic stress responses in plants (Sun et al., 2013). For instance, dehydrin CaDHN1 made great contributions to multiple abiotic stresses in* Capsicum annuum* L. [39]. In potato, the expressions of five* StLEA* genes were induced by both salt and drought stresses [40]. Moreover, the CaLEA1 in pepper acted in ABA signaling regulation, drought, and salt stress responses [41]. In our* KvLEA* expression analysis, the results indicated a similar condition. Its expression is also strongly induced by salt stress under different NaCl concentrations and at different time points. With the increase of the salt concentrations, the responses enhanced gradually and 300 and 400 mM treatment led to obvious responses (Figure 2(b)). On the bases of our previous researches on* K. virginica*, 100 to 200 mM NaCl which was already drastic salt stress for common plant had little effect on it, and only when the concentration reached 300 mM or more did the influence begin to emerge [42–44]. That is why we chose 300 mM for different time gradient treatments and this reflected the strong salt resistance of* K. virginica* at the same time. Under 300 mM condition, the response to NaCl was rapid and the expression level reached high value immediately after 2 h treatment (Figure 2(c)), though, from 2 h to 12 h, the accumulation of the transcripts declined a little, while from 12 h to 24 h the expression rose up again, indicating that there might be a feedback adjustment between the salt stress and the plant resistance. The mix performances were also discovered in previous research on other species under the same conditions. For instance, the* ZmLEA3* in maize displayed two peaks during 48 h treatment. The accumulation reached a peak at 24 h and then reduced from 24 h to 36 h, while from 36 h to 48 h the accumulation rose up again, reaching a new peak [10]. In tobacco, the same presentation also appeared, while the difference was that the mix performances were downregulated [45]. Therefore, more in-depth studies are needed to uncover the complicated mechanisms underlying the* KvLEA* gene and the salt resistance in* K virginica*.

The responses of* KvLEA* gene to abiotic stresses showed us that it could be induced by a variety of abiotic stresses, while the response of* KvLEA* to drought stress was weak (Figure 3(a)). We speculated that there may be other LEA proteins in charge of responding to drought stress in* K. virginica* and there may also be many other LEA proteins having key functions in various abiotic stresses. In addition, according to the gene expression analysis, the gene also responded to heat (Figure 3(b)) and chilling (Figure 3(c)). Although many of the reported LEA genes were discovered to be expressed highly in seeds, there were several LEA genes responding to temperature fluctuation. For example, the* Arabidopsis* LEA genes At2g42530 and At2g42540 were cold responsive genes; thus, they were called COR15A (cold responsive 15A) and COR15B [46].

The most amusing performance appeared in ABA treatment (Figure 3(d)). ABA is a key phytohormone which can be involved in kinds of abiotic stresses. On the one hand, the abiotic stresses such as salt, drought, and cold stresses can lead to the production of ABA. On the other hand, the accumulated ABA can in return affect the expressions of many abiotic responsive genes and lead to stress responses [47, 48]. In this study, the exogenous applied ABA caused an obvious accumulation to this gene (Figure 3(d)). Furthermore, the expression of this gene reached more than 40-fold increase after 6 h treatment, illustrating that there may be complicated correlations between* KvLEA* gene and ABA in* K. virginica*. Lately, a proteomic analysis on maize seeds revealed that the deficiency of ABA in ABA-deficit mutant decreased the accumulation of several LEA proteins [49]. And the transgenic* Arabidopsis* overexpressing pepper* CaLEA1* gene exhibited supersensitivity to ABA [41]. Therefore, more researches are needed to uncover the specific correlation between ABA and* KvLEA* in* K. virginica* in the future. Importantly, transgenic technology should be employed to study the functions of the gene in depth.

## 5. Conclusion

In conclusion, our present study isolated, cloned, and identified the first LEA gene from* K. virginica*. The deduced amino acid sequences of this gene showed that it was a typical LEA protein possessing many common properties of LEA proteins and had high homology with* Arabidopsis* LEA_4 genes At2g36640 and At3g53040. The tissue expression pattern analysis showed that this gene displayed a higher expression level in root. In spite of that, most LEA genes are induced by desiccation and large amount of LEA proteins is synthesized to improve desiccation tolerance, while the LEA genes also have many other known and unknown important functions. Our research revealed that* KvLEA* gene was induced not only by salt stress but also by a variety of other abiotic stresses especially for ABA treatment in* K. virginica*.

## Supplementary Material

In order to verify the BLASTp results, both InterProScan 5 and TMHMM software were aplied to carry out the motif analysis and transmembrane domain pridiction. The two results which were listed in supplemental S1 and S2 also indicated this protein should be a member of LEA_4 group. Therefore, the 18 LEA_4 proteins in Arabidopsis were used for phylogenetic analysis. The gene IDs and names of the 18 proteins were listed in Supplemental S3 and the analysis result was displayed in Fig 1(d).
