# Supplementary material for: KvLEA, a New Isolated Late Embryogenesis Abundant Protein Gene from Kosteletzkya virginica Responding to Multiabiotic Stresses
Source: Biomed Res Int. 2016 Mar 30;2016:9823697. doi: 10.1155/2016/9823697 (PMC4829701; doi:10.1155/2016/9823697)
Supplement: Supplementary file 1 — In order to verify the BLASTp results, both InterProScan 5 and TMHMM software were aplied to carry out the motif analysis and transmembrane domain pridiction. The two results which were listed in supplemental S1 and S2 also indicated this protein should be a member of LEA_4 group. Therefore, the 18 LEA_4 proteins in Arabidopsis were used for phylogenetic analysis. The gene IDs and names of the 18 proteins were listed in Supplemental S3 and the analysis result was displayed in Fig 1(d). [file 9823697.f1.pdf]

Supplementary figures:

S1 Figure.

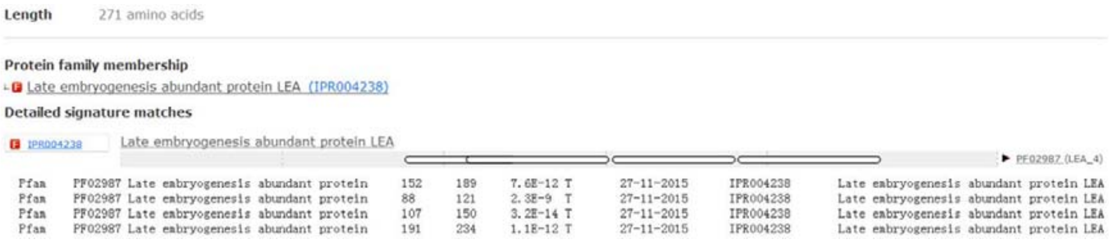

The motif analysis by InterProScan 5 (http://www.ebi.ac.uk/Tools/pfa/iprscan5/). The analysis result showed that there were four repeats of LEA_4 (Pfam02987) motif in the predicted KvLEA protein.

S2 Figure.

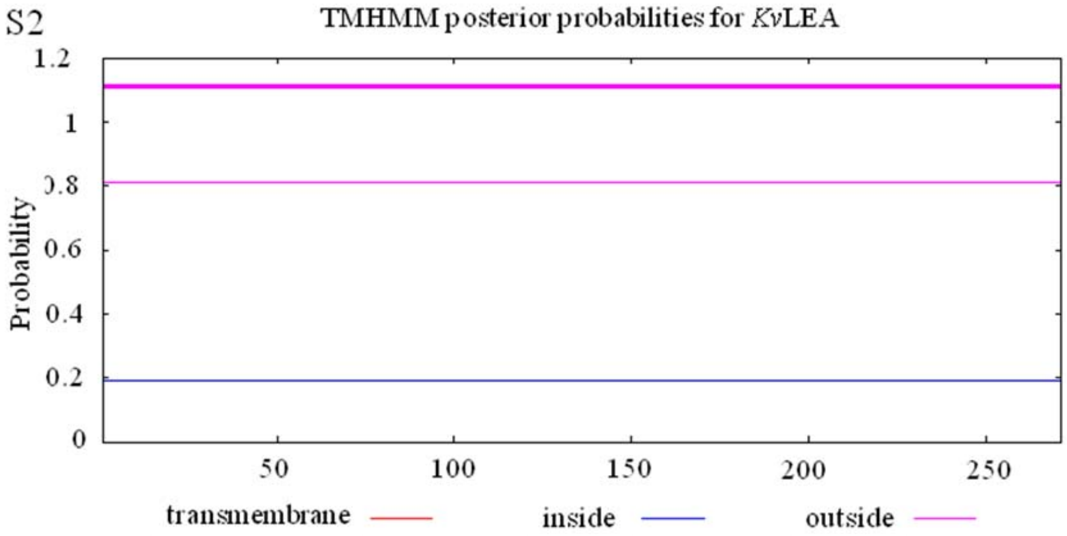

Transmembrane domain pridiction was carried out with TMHMM software (http://www.cbs.dtu.dk/services/TMHMM/). The red line on the above represents transmembrane, blue line below represents inside and pink line in the middle of the two represents outside.

Supplemental table S3

S3 Table. The gene IDs and names of the 18 LEA_4 proteins in Arabidopsis

Gene Name	Gene ID	Gene Name	Gene ID	Gene Name	Gene ID
At1g52690	841701	At2g42530	818853	At3g53040	824470
At1g72100	843541	At2g42540	818854	At4g13230	826939
At2g03740	814901	At2g42560	818856	At4g13560	826989
At2g03850	814911	At3g02480	821185	At4g21020	827849
At2g18340	816349	At3g15670	820810	At4g36600	829812
At2g36640	818236	At3g17520	821017	At5g44310	834454
